# Effects of Nurse-Led Intervention Programs Based on Goal Attainment Theory: A Systematic Review and Meta-Analysis

**DOI:** 10.3390/healthcare9060699

**Published:** 2021-06-09

**Authors:** Bom-Mi Park

**Affiliations:** Department of Nursing, Konkuk University Glocal Campus, Chungju-si 27478, Korea; spring0317@hanmail.net; Tel.: +82-43-840-3960

**Keywords:** nurse, goals, nursing theory, systematic review, meta-analysis

## Abstract

This study aimed to evaluate the effects of the goal attainment theory-based nurse-led intervention programs using a systematic review and meta-analysis. Randomized and non-randomized controlled trials, published from January 2001 to December 2020, were examined using four international databases and four domestic databases. The search, selection, and coding were performed independently by two researchers. R version 4.0.3 and Review Manager (version 5.3) were employed for meta-analysis and quality assessment, respectively. Of the 7529 articles retrieved, 18 were selected for analysis. The random overall effect size of the programs was 0.77 (95% CI = 0.61–0.94). Effect size by dependent variables were 2.36 (95% CI = 0.91–3.82), 1.25 (95% CI = 0.66–1.83), 0.83 (95% CI = 0.55–1.10), 0.64 (95% CI = 0.39–0.89), and 0.58 (95% CI = 0.30–0.85) for interpersonal, cognitive, health behavior, psychological, and indicators of physical health, respectively. Effect size by independent variables were 1.25 (95% CI = 0.86–1.64), 0.76 (95% CI = 0.48–1.03), 0.72 (95% CI = 0.37–1.06), 0.35 (95% CI = 0.21–0.49), and 1.35 (95% CI = −0.15–2.85) for prevention, health promotion, counseling and education, goal-setting and health contract, and parent participation programs, respectively. The effect size by control variables was 1.72 (95% CI = 0.88–2.56) at age ≤17, 0.85 (95% CI = 0.54–1.15) at time (min) 61–90, 1.04 (95% CI = 0.76–1.32) at sessions seven to eight, and 0.93 (95% CI = 0.66–1.19) at duration (weeks) five to eight. Thus, these programs were effective in improving various health aspects. Additionally, they can be recommended in various settings. Because efficacy is also influenced by control variables, considering treatment designs based on intervention characteristics and methodological approaches is warranted.

## 1. Introduction

### 1.1. Background

The exploration of the theory of nursing is not only essential for improving the usefulness of the theory and continuing the academic development, but the evaluation of the theory is also helpful in nursing practice [1]. Moreover, as the use of these theories and the increase in nursing research based on the conceptual framework contributes to the establishment of nursing knowledge, it is important to invigorate their verification for application [2]. Therefore, there must be a continuous development of nursing theories that explain the associated phenomena based on research, and an environment that encourages these that apply them. Furthermore, institutional advancements are also needed [3].

Nurses improve patient’ well-being and the quality of nursing care through communication, a key element of interaction [4]. In addition, during the decision-making process, seamless interaction between the two is an essential element to ensure that appropriate individual nursing services are provided to the patients [5], as they consider their interaction with nurses to be crucial [6]. 

Patients have stated that they experience an increase in the satisfaction and trust in the treatment if they feel respected and gain an understanding of their health status and treatment process through sufficient conversations and clarifications [7]; consequently, this significantly impacts nursing performance [8]. Effective communication in particular increase the quality of nursing service by enabling the formation of a therapeutic relationship with the patient [9]. 

King’s goal attainment theory identifies problems through the communication between nurses and care recipients and sets goals that must be achieved reciprocally [10]. According to this theory, nursing enables action, reaction, and interaction between the nurse and the care recipient by sharing information about their perceptions in the nursing situation; it enables the two parties to recognize specific goals, issues, or problematic situations through communication with a clear purpose [10]. 

The main concepts of the goal attainment theory (1981) comprise identifying a problem through actions, reactions, or interactions, mutual goal setting, seeking ways to achieve the goal, agreement on the method to accomplish it, and transaction and goal attainment [11]. Attaining this goal includes four main elements: (1) Health is achieved through appropriate patient-nurse relationships; (2) nurses and patients must have a mutual understanding of each other; (3) their goals and functions need to be in line with each other; and (4) a nurse needs to use his or her knowledge wholly to establish the relationship and set goals [12,13]. The goal attainment theory pursues objectives within the framework of three interactive systems, namely, personal, interpersonal, and social. The concepts of the personal system are perception, self, growth and development, body image, space, and time. The interpersonal system includes interaction, communication, transaction, roles, and stress. The notions of social systems are organization, authority, power, status, and decision-making [10]. In this regard, the theory represents respect for patients and has a strong emphasis on information exchange, goal-setting, and patient-centered treatment. Therefore, applying King’s goal attainment theory in nursing settings is expected to strengthen the foundation of nursing [14]. 

The examination of recent studies in Korea and overseas has indicated that the goal attainment theory has been reflected in researches on the relationship between patients and nurses [12], nursing practice [14], nursing role [15], mentoring tools for nursing educators [16], telenursing practice [17], and nursing interventions [18,19].

Intrinsically, as a result of reviewing the nurse-led intervention programs based on King’s goal attainment theory, we found that in a study of arthritis patients, the body mass index (BMI), which was the same dependent variable, appeared to have no significant decrease [20]. This was contrary to a research on female college students in their twenties [21] that reported a significant decline. However, it is difficult to determine the most appropriate configuration method for the control variable as factors such as age, time, session, and duration varied in each study. Therefore, it is necessary to comprehensively examine each individual research study to obtain objective and valid results. 

Although nurse-led intervention programs based on the goal attainment theory have been steadily increasing in recent years, it is difficult to draw general conclusions on their efficacy due to the dynamic research subjects, operation methods, and contents. Therefore, to construct such programs that are effective, it is essential to systematically analyze the various studies conducted thus far. Therefore, this study aimed to contribute to the improvement of evidence-based nursing practice by systematically analyzing the effect of nurse-led intervention programs based on the goal attainment theory conducted in various environments in Korea and overseas. 

### 1.2. Purpose

The purpose of this study was to systematically review nurse-led researches based on the goal attainment theory to confirm their general characteristics, and to conduct meta-analysis in order to comparatively analyze the nurse-led programs’ overall mean effect size of the dependent variables, the effect size by the types of dependent variables, the effect size by types of the independent variables, and the effect size by the types of control variables.

### 1.3. Definitions of Terms

#### Systematic Review and Meta-Analysis 

Systematic review is an overview of primary literature with a firm research objective and method according to a clear and reproducible methodology. Meta-analysis is a statistical technique for integrating the results of two or more studies to form a comprehensive inference and is performed to evaluate the effectiveness of interventions [22]. In this study, for the nurse-led intervention program to which the goal attainment theory was applied, the studies were selected according to the criteria suggested in the systematic review and a meta-analysis was performed using the statistical values of each study.

## 2. Research Method

### 2.1. Study Design

This systematic review and meta-analysis study were conducted to integrate and analyze nursing intervention program research based on the goal attainment theory in Korea and overseas. 

### 2.2. Data Selection Criteria

This study was conducted in accordance with the systematic review handbook of the Cochrane collaboration [23] and the systematic review reporting guidelines [24] proposed by the Preferred Reporting Items for Systematic Reviews and Meta-Analysis group. For the literature selection, core questions regarding the goal attainment theory-based nursing intervention programs were first established (participants, interventions, comparisons, outcomes, and study design). This was followed by the process of searching domestic and international electronic databases in accordance with the selection and exclusion criteria. In addition, literature search requires a manual search outside of a database, and the manual search uses snow balling to review references cited from literature obtained through and electronic database [22]. Studies published in journals and doctoral dissertations were included.

#### 2.2.1. Selection Criteria

1. Participants

There were no restrictions on the selection of the study participants. All studies that involved nursing interventions based on the goal attainment theory were included as subjects in this study.

2. Interventions

This study examined nursing interventions based on the King’s goal attainment theory. Nursing with goal attainment theory, nursing with goal attainment, and nursing with King’s theory were included. If there was no mention of King, those that applied the concepts of the goal attainment theory were included.

3. Comparisons

The comparisons were made with the group that did not receive the goal attainment theory-based nursing interventions.

4. Outcomes

After the implementation of the specified programs, the suggested outcome variables were selected.

5. Study design

The study types included nurse-directed randomized (RCTs) and non-randomized controlled trials (NRCTs). 

#### 2.2.2. Exclusion Criteria

Simple goal achievement or goal-setting nursing interventions that were not based on King’s goal attainment theory were excluded. Additionally, language other than Korean and English were omitted as well. Studies that were not experimental design (e.g., meta-analysis, survey research, qualitative studies, etc.), one group experimental design, and studies without original were excluded.

### 2.3. Ethical Considerations

The ethical approval for this research process was exempted by the Institutional Review Board of the affiliated institution (KKUIRB-7001355-202101-E-131).

### 2.4. Data Search and Selection

#### Data Search and Selection

The data search was conducted on Korean and international journals and doctoral dissertations published in the last 20 years, from 1 January 2001 to 31 December 2020. It was restricted to the Korean or English literature. Prior to the literature search, keywords for each database were selected based on the core question, and a search strategy was established using the MeSH and text words. The domestic search engines employed were the Research Information Sharing Service (RISS), ScienceON, the Korean Studies Information Service System (KISS), and the Korean Association of Medical Journal Editors (KoreaMed). The international database search engines used were EMBASE, PubMed, CINAHL, and Cochrane Library. In addition, after conducting an online search through the search databases, a list of references was manually examined. 

The research terms were chosen using MeSH term and text word. The search terms were (“goal attainment” OR “goal attainment theory” OR “goal attainment AND nursing [MeSH]” OR “goal attainment theory AND nursing [MeSH]” OR “King’s theory AND nursing [MeSH]” OR “Kings theory AND nursing [MeSH]”).

Throughout the data collection and the selection process, two researchers independently reviewed all the studies included in the analysis. In the case of a disagreement, the research was reviewed collectively according to the data selection or exclusion criteria until a consensus was reached. First, a list was created for the literature searched through the database, and any duplicated studies were removed. End Note X7, a bibliographic export program, was employed to identify replicated literature. After eliminating duplicates, only titles or abstracts were assessed to examine if the literature fit the selection criteria. When it was difficult to determine whether a study satisfied the selection criteria only by the title or the abstract, the researchers referred to the contents of the paper to make a decision regarding its selection. Bibliographic information of all documents were managed equally, and records were made by stages for the excluded literature. Those selected in the final stage were recorded on a coding table through extraction of data on the author, year, researcher, research design, participant classification, number of participants, their average age, number of participants by age group, place of intervention, country of intervention, type of publication, country of publication, type of intervention program, frequency of intervention, and results.

### 2.5. Data Analysis

#### 2.5.1. Quality Evaluation of Each Study

The quality evaluation of the study was conducted to assess the bias, indicating the degree of risk that can occur when deviating from the true value. In this study, the Cochrane risk of bias (ROB) tool for quality assessment was used for RCTs. For NRCTs, the Cochrane’s Risk of Bias In Non-Randomized Studies of Interventions (ROBINS-I) tool was employed. Quality evaluation was performed for both types of studies after confirming the original text of the manuscript, in accordance with the evaluation guidelines. 

In RCTs, the risk of bias examined by the contents described in each item were classified into “unclear risk”, “low risk”, or “high risk”. In NRCTs, it was evaluated as “no information”, “low”, “moderate”, “serious”, “critical”. The criteria for assessing the quality of the study were applied to the results, which were then entered into the RevMan.

#### 2.5.2. Verification of Publication Bias

To understand the bias that occurs when there is a relationship between the statistical significance of research results and publishability, publication bias was checked using a funnel plot. If the funnel-shaped plot is visually symmetric, it indicates a decrease in the possibility of publication. However, if it is asymmetric, the probability may increase. Moreover, as an analysis method for objective verification, Egger’s linear regression asymmetry test was conducted for verification [25]. Furthermore, the trim-and-fill method was employed to reveal the extent of the impact of publication errors on the results of the study [26].

#### 2.5.3. General Characteristics of the Studies

The general characteristics of nursing intervention programs based on the goal attainment theory were analyzed using coding tables.

#### 2.5.4. Calculation of the Effect Size of the Intervention

A meta-analysis was performed using the R software version 4.0.3 to determine the effect size of the interventions in the 18 papers at the final stage of selection. To identify the effect of the goal attainment theory-based nursing interventions, the mean and standard deviation values after the experiment were analyzed under the premise that they were the same for both the experimental and control groups. The standardized mean difference value outlined in the results was interpreted as the effect size. According to Cohen’s interpretation of the effect size, it was interpreted as small, medium, and large if it was 0.20, 0.50, and 0.80 or above, respectively [27].

To analyze the heterogeneity of each study, a homogeneity test was performed using Higgin’s I^2^ index. Assuming that each research had its own effect, calculations were performed using a random effects model that reset the weight because the homogeneity of the effect size was not secured [28,29]. The statistical significance of the effect size was determined by the 95% confidence interval (CI) in the overall effect test; the significance level was set at 5% [30].

### 2.6. Research Model

This study was based on the personal and interpersonal systems proposed by King. A model was constructed that was tailored to suit this research. This was done by including the indicators of physical health, health behavior, psychological, and psychological programs suggested in a meta-analysis study of the self-determination theory-based interventions [31] as well as by selecting the cognitive and interpersonal programs. This was followed by the grouping of independent and dependent variables using the categorization method suggested in a previous study [32]. 

**Figure 1:**
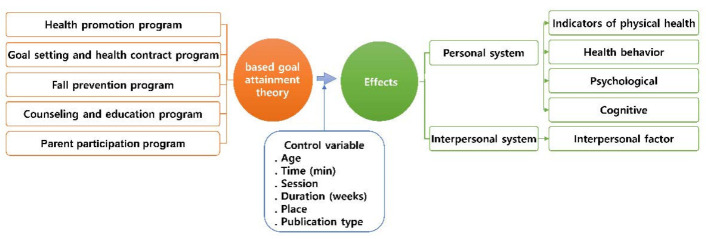
Research model.

The independent variables were classified into the following programs: health promotion, goal-setting and health contract, fall prevention, counseling and education, and parent participation. The dependent variables were categorized into personal (indicators of physical health, health behavior, psychological, and cognitive) and interpersonal systems (interpersonal). The moderating variables were age, time, session, duration (weeks), place, and publication type. The model of this study is shown in Figure 1.

## 3. Results 

### 3.1. Data Selection

The domestic search engines RISS, ScienceON, KISS, and KoreaMed provided 553, 320, 194, and 48 cases respectively. The international database search engines EMBASE, PubMed, CINAHL, and Cochrane library provided 646, 311, 115, and 283 cases, respectively. Therefore, overall 2470 cases were searched and two other references were added by using snow balling from literature obtained through and electronic database. As a result, total 2472 papers were selected. 

**Figure 2:**
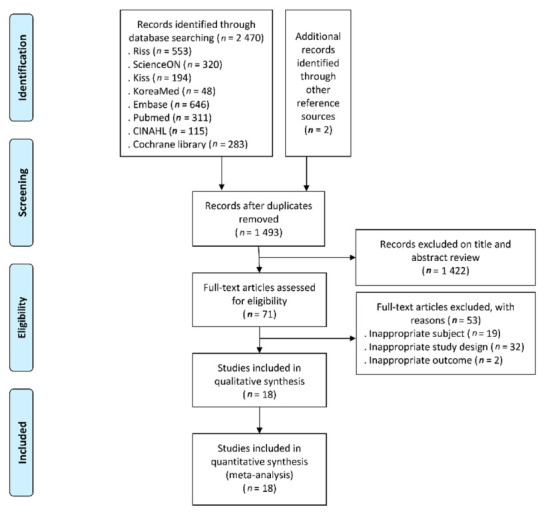
Flow diagram of study selection.

After excluding duplicates, 1493 studies remained, of which 1422 were excluded based on the title and abstract review. After reviewing the remaining 71 articles, we eliminated 53 for the following reasons: inappropriate subjects, improper study designs, and inappropriate outcomes for 19, 32, and two articles, respectively (Figure 2). Finally, 18 papers were selected (*n* = 18, References [18,19,20,21,33,34,35,36,37,38,39,40,41,42,43,44,45,46]).

### 3.2. General Characteristics

The characteristics of the final 18 research papers are shown in Table 1. In terms of the year of publication of the analyzed studies, three (16.7%) were reported from 2001 to 2010, 15 (83.3%) from 2011 to 2020, and six (33.3%) in 2019 alone, which accounted for the largest number of research studies. Regarding the nationality of the lead authors, Korea reported the most studies with 17 cases (94.4%), while Thailand reported one (5.6%) in 2020. With respect to the research design, six cases were RCTs (33.3%), while 12 were NRCTs (66.7%). The number of participants in the studies ranged from a minimum of 17 to 64. Furthermore, four papers (22.2%) were doctoral theses, while 14 (77.8%) were reported in academic journals.

### 3.3. Quality Evaluation Result

**Figure 3:**
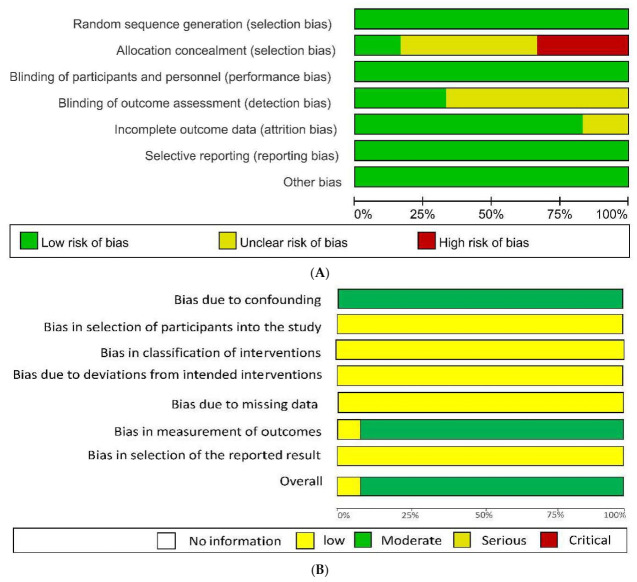
Risk of bias. (**A**) Risk of Bias assessment tool for randomized study graph, (**B**) risk of bias assessment tool for non-randomized study graph.

The characteristics of the risk assessment of bias in the literature used in this study are as follows (Figure 3). As a result of using Cochrane’s ROB tool in six of the 18 selected papers, RCT articles were evaluated as “low risk” when random sequence generation was used. When allocation concealment was examined, one, three, and two cases was identified as “low risk”, “unclear risk”, and “high risk”, respectively. The three studies evaluated as “unclear risk” did not mention blinding, and that assessed as “high risk” used random numbers and odd-even methods; however, there was no blinding. When the “blinding of participants and personnel” were mentioned, all six of the cases were considered to be “low risk”. When blinding of outcome assessment was conducted, two and four cases were evaluated as “low risk” and “unclear risk”, respectively; moreover, there was no mention of blinding the evaluator. In the case of incomplete outcome data, five cases were evaluated as “low risk”, and 1 as “unclear risk”. Furthermore, one case did not mention the reason for elimination. When there was selective reporting, all six cases were assessed to be “low risk”. Lastly, other biases were evaluated as “low risk” in all six cases.

Regarding the results of using Cochrane’s ROBINS-1 for the 12 NRCT papers, in the case of bias due to confounding and absence of its mention, all 12 articles were evaluated as “moderate risk”. They were assessed as “low risk” in the case of bias in selection of the study participants, classification of interventions, bias due to deviations from intended interventions, and those due to missing data. For bias in measurement of outcomes, one case was evaluated as “low risk”, as it was conducted by a research assistant who did not participate in the research intervention. The remaining 11 cases were considered as “moderate risk” because there was no blinding in the measurement of the results. For bias in selection of the reported result, all 12 cases were evaluated as “low risk”. Overall, one case had a bias for confounding; however, it was assessed as “low risk” because the results were measured by a research assistant. The outstanding 11 cases were evaluated as “moderate risk”, because there was no blinding for confounding variables and the results’ measurement. 

### 3.4. Effect Size

#### 3.4.1. Overall Mean Effect Size

The average effect size of the goal attainment theory-based programs was calculated using the effect size and standard deviation from 88 dependent variables of the 18 papers analyzed in this study. Since individual research studies were conducted independently and the samples and intervention methods of the studies were different, this study assumed that the effect size of the population was not homogeneous and acknowledged the variance between the studies. Furthermore, to generalize and apply the research results to other groups, the average effect size was calculated by applying the random-effects model. The average effect size of the program based on goal attainment theory was 0.77 (95% CI = 0.61–0.94), which, according to Cohen’s effect size classification, can be interpreted as a medium size; it was found to be statistically significant. The 88 papers included in the meta-analysis were examined for heterogeneity in the effect size. Consequently, a large degree of heterogeneity was identified with a Q value of 620.99 (*p* < 0.001), and an I^2^ value of 86.0% (Table 2).

#### 3.4.2. Effect Size According to the Dependent Variable

**Figure 4:**
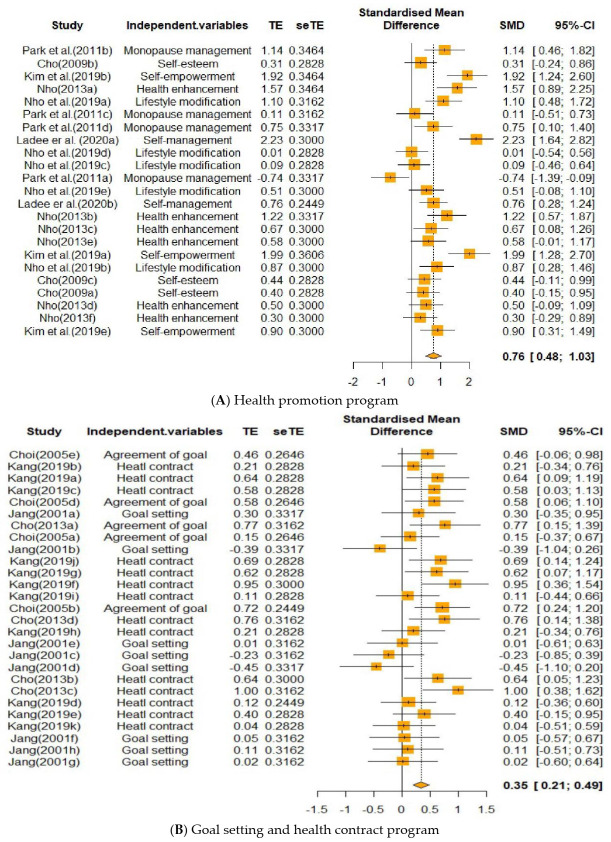

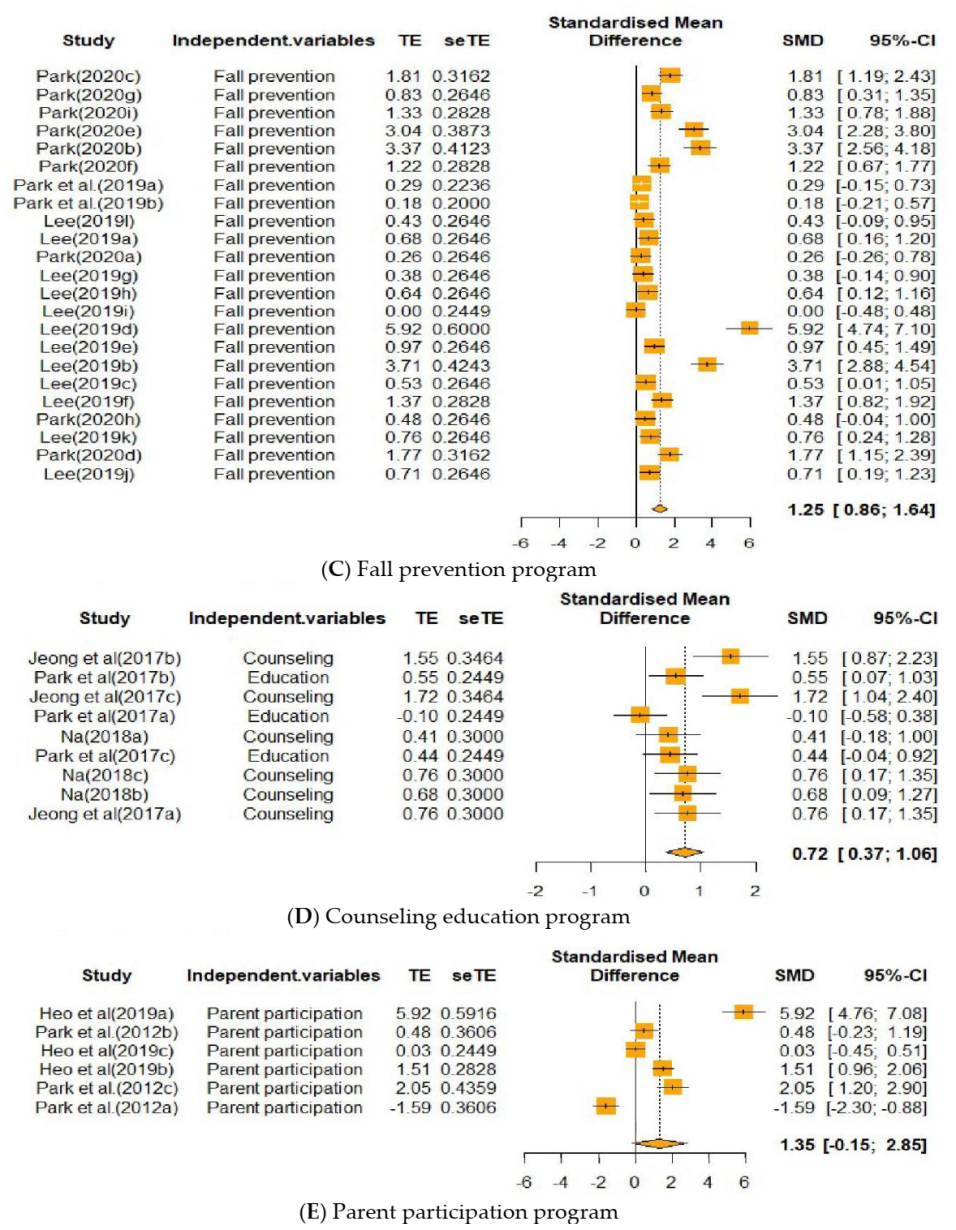
Forest plot of the effect of independent variables: (**A**) Health promotion program, (**B**) Goal setting and health contract program, (**C**) Fall prevention program, (**D**) Counseling education program, (**E**) Parent participation program.

In terms of the intervention types, the contents of each of the 18 papers were comparatively analyzed and classified into five subgroups. The specific outlines of the intervention types are as follows: (1) Health promotion program, comprising six cases: self-management behavior, self-empowerment intervention, multidisciplinary lifestyle modification, integrative self-esteem improvement, sexual health enhancement, and integrated menopause management; (2) goal-setting and health contract program, consisting of four cases regarding agreement of goal attainment method, mutual goal-setting nursing intervention, health contract intervention, and antioxidant improvement program with health contract; (3) fall prevention program, comprising three cases; (4) counseling and education program, which included three cases; and (5) parent participation program, consisting of two cases (Figure 4).

As presented in Table 2, the cases of programs based on the goal attainment theory according to the dependent variables are as follows, from the highest in the number of cases to the lowest: Indicators of physical health (*k* = 32), health behavior (*k* = 24), psychological (*k* = 24), interpersonal (*k* = 5), and cognitive (*k* = 3). The effect size was as follows: interpersonal (*ES* = 2.36), cognitive (*ES* = 1.25), health behavior (*ES* = 0.83), psychological (*ES* = 0.64), and indicators of physical health (*ES* = 0.58). Interpersonal, cognitive, and health behavior variables showed a large effect size, while psychological and indicators of physical health variables demonstrated a medium effect size; they were statistically significant at a 95% confidence interval. The difference in effect size was found to be statistically significant, as the *Q*_b_ value was 9.98 (*df* = 4, *p* < 0.05). 

#### 3.4.3. Effect Size According to the Independent Variable

**Figure 5:**
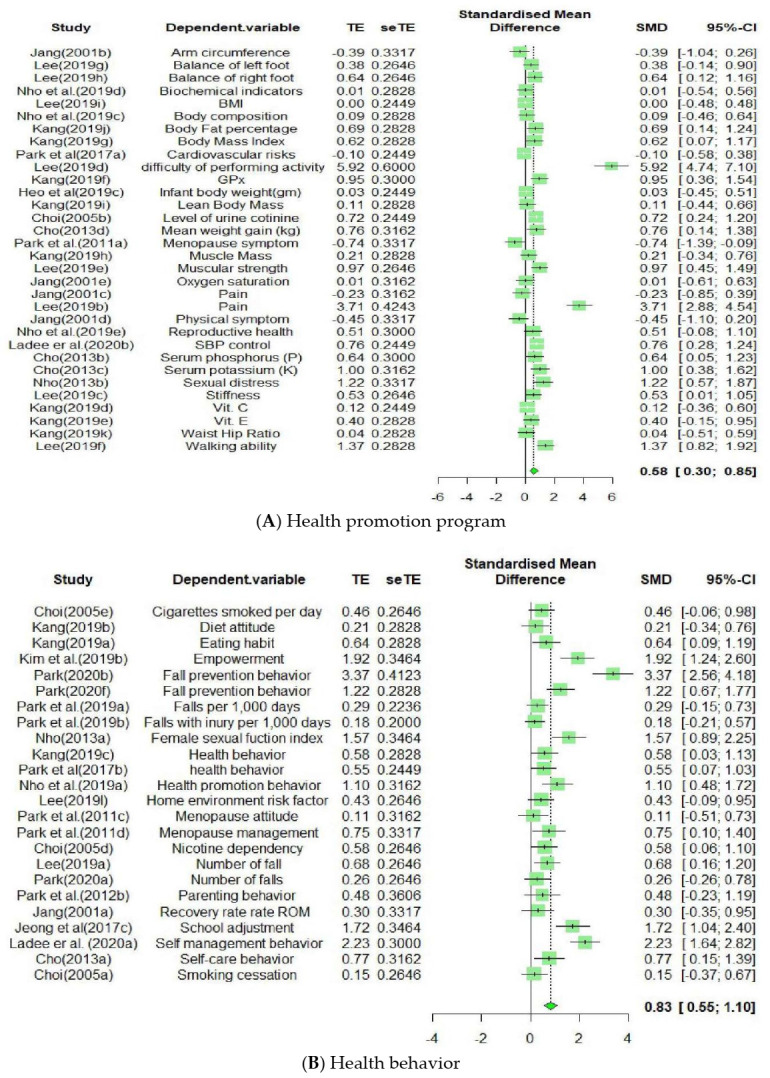

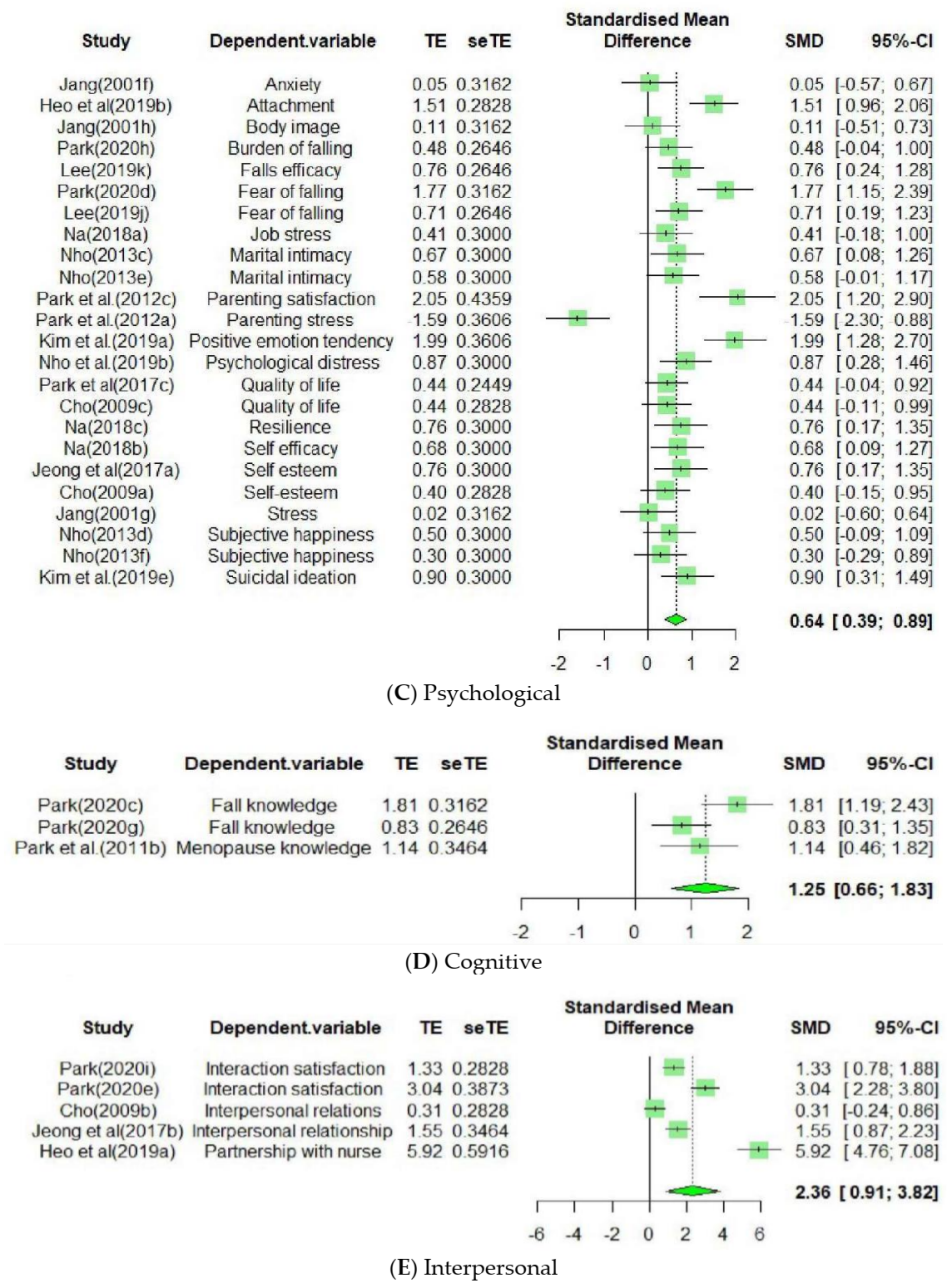
Forest plot of the effect of dependent variables: (**A**) Indicators of physical health, (**B**) Health behavior, (**C**) Psychological, (**D**) Cognitive, (**E**) Interpersonal.

When the program effects were measured, 88 main outcome variables were identified. As a result of their comparative analysis, they were classified into five subgroups. The outcome variables were classified into 32, 24, 24, three, and five variables for the indicators of physical health, health behavior, psychological factors, cognitive factors, and interpersonal factors, respectively (Figure 5). 

As shown in Table 2, the list of the types of programs based on goal attainment theory from the highest to the lowest number of cases was as follows: Goal-setting and health contract (*k* = 27), fall prevention (*k* = 23), health promotion (*k* = 23), counseling and education (*k* = 9), and parent participation (*k* = 6). 

The effect size of the programs was as follows, in the order of the largest to the smallest: Fall prevention (*ES* = 1.25), health promotion (*ES* = 0.76), counseling and education (*ES* = 0.72), and goal-setting and health contract (*ES* = 0.35). The effect size of the parent participation program was 1.35; however, the confidence interval was not statistically significant, including the 0 value. The fall prevention program showed a large effect size, while the health promotion, counseling and education, and goal-setting and health contract programs demonstrated a medium effect size; they were statistically significant at the 95% confidence interval. The *Q*_b_ value of the difference in effect size was 24.50 (*df* = 4, *p* < 0.001), which was found to be statistically significant.

#### 3.4.4. Effect Size According to the Control Variable 

The heterogeneity of the effect size between the studies of the goal attainment theory-based programs was large; therefore, the results of analyzing the control variables to explain the background for this have been outlined in Table 2. The number of cases, from highest to lowest in terms of age, were as follows: 18–59 years old (*k* = 47), 60 years or older (*k* = 23), mixed age (*k* = 10), and 17 years old or younger (*k* = 8). The largest effect size was observed in the age group of 17 years or younger (*ES* = 1.72), followed by those aged 60 years or older (*ES* = 1.25), mixed age (*ES* = 0.85), and 18–59 years of age (*ES* = 0.39). The effect size, by large, in the age groups of 17 years old or younger, 18–59 years old, the mixed age group, and that between 18–59 years had a medium effect size that were all statistically significant at the 95% confidence interval. The difference in the effect size appeared to be statistically significant, as the *Q*_b_ value was 27.06 (*df* = 3, *p* < 0.001).

Regarding time in minutes, the number of cases, from the highest to the lowest, were 31–60 min (*k* = 29), 61–90 min (*k* = 26), 30 min or less (*k* = 20), and 91–120 min (*k* = 13). The effect size was the largest at 61–90 min (*ES* = 0.85), followed by 31–60 min (*ES* = 0.81), 30 min or less (*ES* = 0.70), and 91–120 min (*ES* = 0.64). The effect size according to the time (min) was large for 61–90 min and 31–60 min, and medium for 30 min or less and 91–120 min; the results were statistically significant at the 95% confidence interval. The difference in the effect size with a *Q*_b_ value of 0.76 (*df* = 3, *p* > 0.05) was not statistically significant.

For the sessions, the number of cases have been listed in the order of the highest to the lowest, being seven to eight (*k* = 42), four or less (*k* = 22), nine or more (*k* = 20), and five to six (*k* = 4). The effect size was largest in seven to eight sessions (*ES* = 1.04), followed by five to six (*ES* = 0.84), nine or more (*k* = 20), and four or less (*ES* = 0.43). The effect size by sessions demonstrated that the seven to eight and five to six sessions had a large effect size, while nine or more and four sessions or less had a medium effect size; the findings were statistically significant at the 95% confidence interval. The difference in effect size was found to be statistically significant with a *Q*_b_ value of 12.74 (*df* = 3, *p* < 0.01) 

For the duration (weeks), the number of cases, from the highest to the lowest, were as follows: five to eight (*k* = 43), four or less (*k* = 24), nine to 12 (*k* = 18), and 13 or more (*k* = 3). The effect size was largest at five to eight weeks (*ES* = 0.93), followed by four weeks or less (*ES* = 0.77), nine to 12 weeks (*ES* = 0.54), and 13 weeks or more (*ES* = 0.30). The effect size by duration was large for five to eight weeks, while it was of medium size for four weeks or less and nine to 12 weeks. The results were statistically significant at the 95% confidence interval; however, the duration of 13 weeks or more was not statistically significant as the effect size was small. The difference in the effect size was found to be statistically significant with a *Q*_b_ value of 8.80 (*df* = 3, *p* < 0.05).

The greatest number of cases were at the hospitals (*k* = 47), followed by the communities (*k* = 41). Moreover, the effect size was the largest for the hospitals (*ES* = 0.82), followed by the communities (*ES* = 0.72). The effect size by place was large at the hospitals and medium in the communities; the findings were statistically significant at the 95% confidence interval. The difference in the effect size with a *Q*_b_ value of 0.38 (*df* = 1, *p* > 0.05) was not statistically significant.

Regarding the publication type, the number of cases was the greatest in the doctoral thesis category (*k* = 35), followed by the journals (*k* = 53). The effect size was the largest for the former (*ES* = 0.98), followed by the latter (*ES* = 0.64). The effect size by publication type was large with the former and medium size with the latter. The results were statistically significant at the 95% confidence interval; the difference in the effect size with a *Q*_b_ value of 3.81 (*df* = 1, *p* > 0.05) was not found to be statistically significant. 

These results indicated that the effect of the programs based on the goal attainment theory explained the difference in the magnitude of the effect between the studies by the controlling variables age, session, and duration (weeks). These findings demonstrated that the difference in the effect size of each study regarding the programs was explained by the age, session, and duration (weeks) variables in determining the effect of those programs. Alternatively, the largest effect size of the programs was when the age group was 17 years or younger, there were seven to eight sessions, or the duration was five to eight weeks.

### 3.5. Publication Bias Analysis

**Figure 6:**
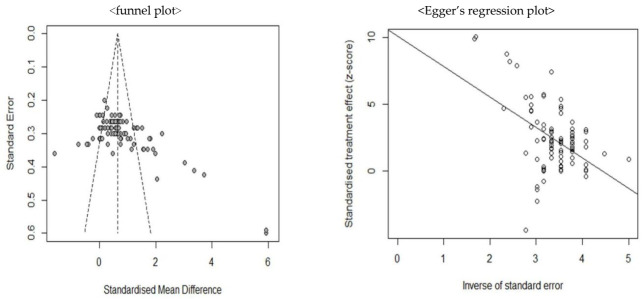
Publication bias analysis.

The results of the publication error analysis to verify the validity of the research results showed slight asymmetry in the upper left and lower right areas of the funnel plot (Figure 6). However, it did not deviate significantly from the left-right symmetry oriented to the central straight line. Nevertheless, the effect size and standard error demonstrated a linear relationship in the Egger’s regression analysis, and the bias was 10.09 (*t* = 6.56, *df* = 86, *p* < 0.001) and appeared to be statistically significant. Therefore, the possibility of publication errors cannot be dismissed.

The results of the trim-and-fill analysis to reveal the extent of the effect of publication errors on the study outcomes are presented in Table 3. When 0 cases that were not reported due to publication errors were added, the corrected effect size was found to be 0.77. This result was the same as when compared to the original effect size of 0.77; as it was statistically significant, it could be considered as a significant result. Therefore, although the publication errors in the studies examined cannot be dismissed, the corrected effect size was also confirmed to demonstrate the same result. Consequently, nursing intervention programs based on the goal attainment theory can be concluded to have a medium effect. 

## 4. Discussion

This study investigated the effects of nurse-led intervention programs, based on the goal attainment theory, through a systematic review and meta-analysis of RCTs and NRCTs that focused on the corresponding effect between 2001 and 2020.

Of the 18 studies included in this systematic review, 15 (83.3%) were published after 2010. The majority, 17 studies (94.4%), were conducted in Korea, while one (5.56%) was carried out in Thailand in 2020. After Jeong and Kim [8] published their study in the Journal Korean Academy Nursing in 2017, there was a rapid increase in cases; the greatest number of studies were reported in 2019 with six articles (33.3%), thus reflecting the increasing interest in the goal attainment theory in recent times. 

In this study, 13 journals and five doctoral theses were selected, resulting in a total of 18 studies. In addition, 88 dependent variables were chosen to calculate effect sizes. Considering the heterogeneity of the individual studies, a meta-analysis was performed using a random-effects model. The overall random effect size of the nurse-led intervention programs based on goal attainment theory was 0.77, and the random effect size corrected by the trim-and-fill was 0.77, depicting a medium effect size. Due to the challenges in discovering studies on the meta-analysis of nursing interventions based on theories, the results were compared with those of a meta-analysis research on the effect of a group counseling program based on Alder’s theory [32]. The random effect size in the previous study was 0.96, reflecting a large effect size. Therefore, it is necessary to consider the use of a program based on theories in the future. In particular, King’s goal attainment theory can be considered as more effective in patient-centered nursing, as the theory enables patients and nurses to set and achieve their goals collectively through interactions [19].

As the participants and programs in this study were diverse, the dependent variables also varied. However, from the systems proposed by King, the effect size for the social system remained unidentified because there were no dependent variables for the social system. Therefore, an intervention study with additional dependent variables for the social system will be needed in the future to propose the effect size for the concepts presented in the theory. 

The results of comparing the effect size according to the program demonstrated that the interpersonal program had the largest size of 2.36, while those of the cognitive variable, health behavior, psychological variable, and indicators of physical health were 1.25, 0.83, 0.64, and 0.58, respectively. The interpersonal variable, having the highest effect size, was the main concept in the goal attainment theory [10] and suggested a direction for nursing intervention programs based it. Therefore, in order to construct a nursing intervention program to improve interpersonal factors in the future, designing a goal attainment theory-based program can be considered as an effective method.

In a meta-analysis of self-care knowledge in a previous study [47], the effect size was 1.08. In the current study, the effect size of the cognitive variables, including knowledge, was 1.25. Although the previous research also indicated a high effect size, it may be insufficient to generalize the individual analysis studies in three to five cases. Therefore, an additional meta-analysis of the cognitive variables, including knowledge, will be needed in the future.

In a meta-analysis study based on the intervention of self-determination [31], the effect size of health behavior was 0.45. In this study, it was 0.83 and medium; it was higher than the effect size of the aforementioned research. Both theories are based on patient-centered nursing. However, the goal attainment theory in particular is considered to have encouraged the supportive role in the process of interaction between nurses and patients as they set goals and achieve them together.

Lee and Park [30] found the total psychological effect size to be 0.30. In a study by Park and Bae [48], as a result of psychoeducational intervention, the positive and negative effect sizes were 0.29, respectively. The research by Ntoumanis et al. [31], which was based on a theory, showed a small effect size of 0.29 with the psychological variable. However, in this study, the psychological effect size was 0.64, indicating a medium effect size, and intervention programs based on the goal attainment theory are expected to demonstrate a positive result on the size of the psychological effect. 

In the study by Ntoumanis et al. [31], the number of dependent variables for indicators of physical health was 16, and the effect size was reported as 0.04, *p* = 0.067. However, in this study, the number of dependent variables was 32 and the effect size was 0.58, indicating a statistically significant effect, despite which, the variables of indicators of physical health may be insufficient to observe the effect in a short-term study. Therefore, a comparative meta-analysis of this period is necessary.

The nurse-led intervention programs based on the goal attainment theory include a wide spectrum of ideas, including six cases of health promotion programs (33.3%), four of goal-setting and health contract programs (22.2%), three of fall prevention programs (16.7%), three of counseling and education programs (16.7%), and two of parent participation programs. Among the statistically significant effect sizes, that of the fall prevention program was large at 1.25. The effect size from a previous meta-analysis on fall prevention was 0.76 [30], reflecting that it was higher in the fall prevention program that applied the goal attainment theory. The intervention of a nurse is vital in preventing a patient’s fall; however, the latter’s participation is crucial as well [19]. Therefore, the effect size is assumed to have been higher by increasing the patient’s participation by setting goals together with the patient. 

The health promotion program showed the next largest effect size, which was medium at 0.76. In a previous study [49] that was not based on theory, it was small at 0.12. In this study, the total effect size of the 23 dependent variables was 0.76. However, direct comparison seemed unreasonable as the dependent variable in a previous study [49] was limited to BMI. Consequently, the previous study was compared with BMI, which was an individual dependent variable in this study. Its two cases were presented, in which the effect size of 0.00 was not statistically significant, and that of 0.62 was statistically significant. Therefore, the overall effect of indicators of physical health was not high because of the characteristics of the variables. However, this research demonstrated the effect of nursing intervention programs based on the goal attainment theory because the effect sizes were higher than that of the previous one.

The counseling and education programs showed a medium effect size of 0.72. In comparison with a meta-analysis effect size, a previous study by Moon [50] reported the counseling program’s effect size as 0.51. Furthermore, the education program’s effect size of the psychological variable in a study by Oh and Choi [47] was 1.24, while that of knowledge and self-nursing was 1.29, indicating a large effect in the counselling program. The present research included three previous studies that simultaneously conducted counseling and education. However, the result of classifying by titles showed a slight difference, as the effect sizes in the former and the latter were 0.41~1.72 and −0.10~0.55, respectively. Correspondingly, nurse-led intervention programs based on the goal attainment theory had a medium effect on counseling and education programs. In particular, as the former indicated a larger effect size, it is expected to be effective when constructing a counseling nursing intervention program based on the goal attainment theory. 

The goal-setting and health contract program had a small effect size of 0.35. The effect size from a previous study [51] on physical functioning and quality of life was small and significant at 0.11 and 0.09, respectively. As both studies reported small effect sizes, further meta-analysis on these programs is warranted.

The parent participation program showed the highest effect size of 1.35; however, it required cautious interpretation because it was not statistically significant. Moreover, verification through future research will be necessary, as the number of cases was small. Nevertheless, in comparison with a previous study on parenting support programs [52], which showed a large and a medium effect size in parenting capacity and social psychology, respectively, the parental participation programs that applied the goal attainment theory indicated a large effect size. Therefore, further meta-analysis of parent participation programs will be needed in the future. 

The control variables were classified into age, time (min), session, duration (weeks), place, and publication type. There was no restriction on the age of participants as the age groups ranged from 34.6 months [18] to 81.38 years [19]. As a result, the effect size was the highest at 1.72 at the age group of 17 years or younger, followed by 1.25 in the age group of 60 years or older. Additionally, the effect size at the mixed age was also large at 0.85. This reflects how the goal attainment theory can be widely used in various age groups, and the findings indicate that such interventions are highly acceptable in practice [53]. 

In the study by Jeon and Park [54], time (min) showed a medium effect size of 0.73, when it was less than 60 min, and a low effect size of 0.37, when it was more than 60 min. In this study, the effect size was medium (0.85 for 61 to 90 min and medium at 0.81 for 31 to 60 min). However, the effect size was small for 91 to 120 min at 0.64, which was consistent with the previous study, showing a lower effect size when the time was prolonged. This reveals the necessity of organizing the time appropriately when constructing an intervention program, based on the results of the meta-analysis. On average, 31–90 min is expected to produce good results. 

In the study by Robroek et al. [49], the five sessions or more effective than five sessions or less. In this study, seven to eight sessions showed a large effect size of 1.04; nine sessions or more was 0.60, and an improved size could not be obtained even when the program was performed several times. Therefore, it is suggested to configure programs into seven to eight sessions when proceeding with interventions in the future.

In this study, the effect size of duration (weeks) was 0.93, 0.54, and 0.30 for five to eight, nine to 12, and 13 weeks or more, respectively, indicating that the number of weeks increased as the effect size decreased. The study by Jeon and Park [54] reported results that were contrary to this study, with 0.59 for 10 weeks or more and 0.40 for less than 10 weeks; however, it was not statistically significant. This study disclosed that long-term persistence does not provide good effects; nevertheless, additional research is needed to explore the duration (weeks). Furthermore, the effect size was compared for place and publication types. Although all results showed medium-sized effects, there were no statistically significant differences. Therefore, repeated research related to place and publication type is warranted in the future. 

This study presented a research model for a nursing intervention program based on King’s goal attainment theory, and the effect sizes of the dependent, independent, and control variables were identified. This research is significant in that it contributes to the improvement of evidence-based nursing practice by presenting scientific verification on the importance of theory-based nursing intervention programs. Despite the significance, it has certain limitations. First, the subjects and types of programs included in the analysis were diverse; hence, the study was unable to investigate the detailed elements. Therefore, a detailed meta-analysis of effective programs will be needed in future studies. Second, comparisons with previous research were not diverse because of the lack of a systematic literature review and meta-analysis of intervention programs based on nursing theories. Therefore, we suggest future research to conduct systematic literature review and meta-analysis studies on intervention programs based on nursing theories.

## 5. Conclusions

The overall effect size identified in this study was 0.77, thus confirming the effect of nursing intervention programs based on the goal attainment theory. In particular, this study conducted a systematic literature review and meta-analysis of intervention programs based on nursing theories, suggesting a positive effect of nurse-led intervention programs founded on theories. As a result of analyzing previous research, the effect size for interaction, which is one of the concepts of goal attainment theory, was the highest at 2.36. This confirmed the effect of the goal attainment theory when constructing a program to improve interactions. In terms of the statistically significant effect size, the fall prevention program showed a large size of 1.25. In addition, it was the highest for the age group of 17 years or younger, seven to eight sessions, and duration (weeks) of five to eight weeks. Nurse-led intervention programs in the future should be configured with relevant factors having high effect sizes based on the results of this study, and the effects should be repeatedly verified to improve their efficacy. The goal attainment theory entails that patients and nurses collectively set and achieve goals through interactions in order to perform patient-centered nursing, and its efficacy has been demonstrated. Therefore, based on these findings, it is anticipated that this theory will be actively used in clinical practice. 

## Figures and Tables

**Table 1:** Characteristics of the included studies [18,19,20,21,33,34,35,36,37,38,39,40,41,42,43,44,45,46].

No	Author (Year)	Researcher	Design	Participants Sample Size (*n*)		Mean Age (Year) or Age: Number of Persons		Research Place /Country	Publication	Intervention Program	Time (Min/Session/Duration (Week))	Outcome
				Exp.	Con.	Exp.	Con.					
1	Park (2020)	Nurse	NRCT	Patients 27 Nurses 28	Patients 30 Nurses 30	Patients 78.78 Nurses 42.45	Patients 78.77 Nurses 35.97	Hospital /Korea	Doctoral dissertation	Fall prevention program (1) Individual training (patients and nurses) (2) Group training (nurses) (3) Individual counseling (patients and nurses) (4) Individual activities (nurses)	20~30/8/8	- Patients 1. Number of falls (-) 2. Fall prevention behavior (↑) 3. Fall knowledge (↑) 4. Fear of falling (↓) 5. Interaction satisfaction (↑) - Nurses 6. Fall prevention behavior (↑) 7. Fall knowledge (↑) 8. Burden of falling (-) 9. Interaction satisfaction (↑)
2	Ladee et al., (2020)	Nurse	RCT	Patients 36	Patients 34	35–59: 12 60 > 23	35–59: 9 60 > 25	Hospital/Thailand	Journal	Self-management behavior program (1) Group small-talk education (2) Individual, remind, reinforce	10~120/5/10	1. Total score (1) Dietary (↑) (2) Physical activity (↑) (3) Medication adherence (-) (4) Avoidance of cardiovascular disease risks (↑) (5) Home blood pressure monitoring (↑) 2. Systolic blood pressure control (↑)
3	Kim and Han (2019)	Nurse	RCT	Patients 21	Patients 25	19–29: 12 30–39: 4 40–49: 4 50–59: 1 60 > 0	19–29: 15 30–39: 4 40–49: 3 50–59: 2 60 > 1	Hospital/Korea	Journal	Self-empowerment intervention program (1) Education (2) Support	8/2/60	1. Positive emotion tendency (1) Subjective well-being (↑) (2) Psychological well-being (↑) (3) Gratitude (↑) (4) Optimism (↑) (5) Self-esteem (↑) 2. Empowerment (1) Self-esteem (↑) (2) Self-efficiency, future mastery (↑) (3) Community activities, autonomy (↑) 3. Suicidal ideation (↓)
4	Nho and Hwang (2019)	Nurse	NRCT	Women 22	Women 24	49.5	48	Community/Korea	Journal	Multidisciplinary lifestyle modification program (1) Group education, physical activity (2) Individual counseling	120/8/8	1. Health promotion behavior (↑) (1) Health responsibility (↑) (2) Physical activity (↑) (3) Nutrition (↑) (4) Spiritual growth (↑) (5) Interpersonal relationships (↑) (6) Stress management (↑) 2. Psychological distress (1) Depression (↓) (2) Anxiety (↓) (3) Stress (↓) 3. Body composition (1) Body weight (↓) (2) Body mass index (↓) (3) Body fat (-) (4) Abdominal fat (-) 4. Biochemical indicators (1) Hemoglobin (-) (2) High density lipoprotein (-) (3) Low density lipoprotein (-) (4) Triglyceride (-) (5) Glucose (-) 5. Reproductive health (↓)
5	Lee (2019)	Nurse	NRCT	Patients 30	Patients 30	77.53	76.27	Community/Korea	Doctoral dissertation	Fall prevention program (1) Group education, physical activity (2) Individual emotional support	Group 70/8/8 Individual 10/16/8	1. Number of fall (↓) 2. Pain (-) 3. Stiffness (↓) 4. Difficulty of performing activity (↓) 5. Muscular strength (↑) 6. Walking ability (↑) 7. Balance of left foot (↑) 8. Balance of right foot (↑) 9. Body Mass Index (-) 10. Fear of falling (↓) 11. Falls efficacy (↑) 12. Home environment risk factor (-)
6	Heo and Oh (2019)	Nurse	RCT	Infants 30 Parents 60	Infants 32 Parents 64	Infants 34.60 month Parents -	Infants 34.97 month Parents -	Hospital/Korea	Journal	Parent participation improvement program (1) Group education (2) Individual education, demonstration	50~60/10/2	1. Partnership with nurse (↑) (1) Reciprocity (↑) (2) Professional knowledge and skills (↑) (3) Sensitivity (↑) (4) Collaboration (↑) (5) Communication (-) (6) Sharing information (↑) (7) Cautiousness (↑) 2. Attachment (↑) 3. Infant body weight (gm) (-)
7	Kang (2019)	Nurse	NRCT	Female college students 25	Female college students 25	20–24: 22 25 > 3	20–24: 23 25 > 2	Community/Korea	Doctoral dissertation	Antioxidant improvement program (1) Walking (2) Antioxidative diet	Group 60/10/10 Individual -/20/10	1. Eating habit (↑) 2. Diet attitude (-) 3. Health behavior (↑) 4. Vitamin C (↑) 5. Vitamin E (↑) 6. Glutathione peroxidase (↑) 7. Body mass index (↓) 8. Muscle mass (-) 9. Lean body mass (-) 10. Body fat percentage (↓) 11. Waist hip ratio (-)
8	Park et al. (2019)	Nurse	NRCT	Patients 52	Patients 45	79.71	81.38	Hospital/Korea	Journal	Tailored fall prevention program (1) Group education, demonstration (2) Individual education, demonstration, counseling	20∼60/6/12	1. Falls per 1000 days (↓) 2. Falls with injury per 1000 days (-)
9	Na (2018)	Nurse	NRCT	Nurses 21	Nurses 23	29.2	29.7	Community/Korea	Doctoral dissertation	Counseling program (1) Group discussion, interview, education (2) Individual counseling, encouraging	100/16/9	1. Self-efficacy (↑) 2. Job stress (-) 3. Resilience (↑)
10	Park, Song, and Jeong (2017)	Nurse	RCT	Patients 32	Patients 32	56.87	55.37	Hospital/Korea	Journal	Tailored education program (1) Group education (2) Individual counseling, encouraging	Group 30/3/30 Individual 15∼20/6/30	1. Cardiovascular risks (1) Cardiovascular disease risk (↓) (2) Total cholesterol (↓) (3) Triglyceride (-) (4) High density lipoprotein-cholesterol (-) (5) Low density lipoprotein-cholesterol (↓) (6) Fasting blood sugar (↓) 2. Health behavior (↑) (1) Health responsibility (↑) (2) Exercise behavior (↑) (3) Healthy diet (↑) (4) Stress management (↑) (5) Smoking cessation (↑) 3. Quality of life (1) Physical quality of life (↑) (2) Mental quality of life (↑) (3) Physical functioning (↑) (4) Role limitations-physical (↑) (5) Role limitations-emotional (↑) (6) Social functioning (↑) (7) Bodily pain (↑) (8) Vitality (-) (9) Mental health (↑) (10) General health (-)
11	Jeong and Kim (2017)	Nurse	NRCT	Middle school students 22	Middle school students 22	14–16: 22	14–16: 22	Community/Korea	Journal	Group counseling program (1) Group discussion, interview, education (2) Individual, counseling, encouraging	Group 45/8/8 Individual 5/16/8	1. Self-esteem (↑) 2. Interpersonal relationship (↑) 3. School adjustment (↑)
12	Cho (2013)	Nurse	RCT	Patients 21	Patients 22	56.52	64.23	Hospital/Korea	Journal	Health contract intervention (1) Self-care behavior performance praise, encouragement, support, self-care log, blood pressure, body weight measurement, exercise, dietary intake diary	30~60/4/4	1. Self-care behavior (↑) (1) Mediation (-) (2) Fistula management (-) (3) Management of physical problems (-) (4) Diet (↑) (5) Exercise and rest (↑) (6) Management of blood pressure and body weight (↑) (7) Social adjustment (↑) 2. Serum phosphorus (P) (-) 3. Serum potassium (K) (↓) 4. Mean weight gain (kg) (↓)
13	Nho (2013)	Nurse	NRCT	Women 42	Women 44	46.71	44	Hospital/Korea	Journal	Sexual health enhancement program (1) Couple discussion, interview, education (2) Individual discussion lecture	90/4/4	- Woman 1. Female sexual function index (↑) (1) Desire (↑) (2) Arousal (↑) (3) Lubrication (↑) (4) Orgasm (↑) (5) Satisfaction (↑) (6) Pain (↑) 2. Sexual distress (↓) 3. Marital intimacy (↑) (1) Cognition (-) (2) Emotion (↑) (3) Sex (↑) 4. Subjective happiness (-) - Husband 5. Marital intimacy (-) (1) Cognition (-) (2) Emotion (-) (3) Sex (-) 6. Subjective happiness (↑)
14	Park and Oh (2012)	Nurse	NRCT	Mothers 19	Mothers 20	30–39: 13 40 > 6	30–39: 13 40 > 7	Community/Korea	Journal	Active parenting program (1) Group discussion, interview, education (2) Individual, discussion lecture	Group 120/8/8 Individual 5/16/8	1. Parenting stress (↓) 2. Parenting behavior (↑) 3. Parenting satisfaction (↑)
15	Park and Lee (2011)	Nurse	RCT	Women 17	Women 20	52.35	55	Hospital/Korea	Journal	Integrated menopause management program (1) Group education (2) Individual support, encouraging	5∼120/24/8	1. Menopause symptom (↓) 2. Menopause knowledge (↑) 3. Menopause attitude (-) 4. Menopause management (↑)
16	Jo (2009)	Nurse	NRCT	Persons 25	Persons 23	<30 5 person 30–39: 15 person 40> 5 person	<30 5 person 30–39: 10 person 40> 8 person	Hospital/Korea	Journal	Integrative self-esteem improvement program (1) Education	60/10/4	1. Self-esteem (↑) 2. Interpersonal relations (↑) 3. Quality of life (↑)
17	Choi (2005)	Nurse	NRCT	Students 29	Students 30	20–29 29 person	20–29 30 person	Community/Korea	Journal	Achievement agreement (1) Education	90/3/2	1. Smoking cessation (-) 2. Level of urine cotinine (↓) 3. Nicotine dependency (-) 4. Cigarettes smoked per day (↓)
18	Jang (2001)	Nurse	NRCT	Patients 20	Patients 17	45.35	43.82	Hospital/Korea	Journal	Mutual goal-setting nursing intervention (1) Education	10∼30/4/1	1. Recovery rate range of motion (↑) 2. Arm circumference (-) 3. Pain 4. Physical symptom 5. Oxygen saturation (-) 6. Anxiety 7. Stress 8. Body image (-)

RCT = randomized controlled trial; NRCT = non-randomized controlled clinical trial; ↑ = statistically significant increase; ↓ = statistically significant decrease; - = no statistically significant difference.

**Table 2:** Effect size and tendency of the variables of nurse-led program based on the goal attainment theory.

A. The Overall Effect Size of Program Based on the Goal Attainment Theory							
Model	*k*	*ES* (g)	95% CI		*Q* (*df*)	I^2^	*p*
			Lower	Upper			
Fixed	88	0.65	0.59	0.71	620.99 (87)	86.0	<0.001
Random		0.77	0.61	0.94			
**B. Effect Size by Dependent Variables**							
Dependent variables	*k*	*ES*(g)	95% CI		*Q*_b_ (*df*)		*p*
			Lower	Upper			
Indicators of physical health	32	0.58	0.30	0.85	9.98 (4)		0.041
Health behavior	24	0.83	0.55	1.10			
Psychological	24	0.64	0.39	0.89			
Cognitive	3	1.25	0.66	1.83			
Interpersonal	5	2.36	0.91	3.82			
Total	88	0.77	0.61	0.94			
**C. Effect Size by Independent Variables**							
Independent variables	*k*	*ES* (g)	95% CI		*Q*_b_ (*df*)		*p*
			Lower	Upper			
Health promotion program	23	0.76	0.48	1.03	24.50 (4)		<0.001
Goal-setting and health contract program	27	0.35	0.21	0.49			
Fall prevention program	23	1.25	0.86	1.64			
Counseling and education program	9	0.72	0.37	1.06			
Parent participation program	6	1.35	−0.15	2.85			
Total	88	0.77	0.61	0.94			
**D. Effect Size by Control Variables**							
Control variables	Subgroups	*k*	*ES* (g)	95% CI		*Q*_b_ (*df*)	*p*
				Lower	Upper		
Age	≤17	8	1.72	0.88	2.56	27.06 (3)	<0.001
	18–59	47	0.39	0.24	0.53		
	≥60	23	1.25	0.86	1.64		
	mix	10	0.85	0.48	1.23		
Time (min)	≤30	20	0.70	0.31	1.10	0.76 (3)	0.869
	31–60	29	0.81	0.55	1.07		
	61–90	26	0.85	0.54	1.15		
	91–120	13	0.64	0.18	1.10		
Session	≤4	22	0.43	0.23	0.62	12.74 (3)	0.005
	5–6	4	0.84	0.03	1.65		
	7–8	42	1.04	0.76	1.32		
	≥9	20	0.60	0.30	0.91		
Duration (Weeks)	≤4	24	0.77	0.41	1.12	8.80 (3)	0.032
	5–8	43	0.93	0.66	1.19		
	9–12	18	0.54	0.32	0.75		
	≥13	3	0.30	−0.10	0.69		
Place	Hospital	47	0.82	0.58	1.06	0.38 (1)	0.538
	Community	41	0.72	0.50	0.94		
Publication type	Doctoral dissertation	35	0.98	0.70	1.25	3.81 (1)	0.051
	Journal	53	0.64	0.44	0.84		

*k* = Number of effect sizes; *ES* = Effect size; CI = Confidence interval.

**Table 3:** Average effect size before and after adjustment for trim-and-fill.

	*k*	*ES* (g)	95% CI		*Q* (*df*)	*p*
			Lower	Upper		
Observed random effect model	88	0.77	0.61	0.94	620.99 (87)	<0.001
Adjusted trim-and-fill model	88	0.77	0.61	0.94	620.99 (87)	<0.001

*k* = Number of effect sizes; ES = Effect size; CI = Confidence interval.

## Data Availability

Not applicable.
